# Evaluation of the Newborn Screening Pilot for Sickle Cell Disease in Suriname Using the Non-Adoption, Abandonment, Scale-Up, Spread, and Sustainability (NASSS) Framework

**DOI:** 10.3390/ijns10030046

**Published:** 2024-07-04

**Authors:** Ming-Jan Tang, Jimmy Roosblad, John Codrington, Marjolein Peters, Aartie Toekoen, Patrick F. van Rheenen, Amadu Juliana

**Affiliations:** 1 Department of Paediatrics, Streekziekenhuis Koningin Beatrix, 7101 BN Winterswijk, The Netherlands; 2 Laboratory Academisch Ziekenhuis, Paramaribo, Surinamejcodrington@azp.sr (J.C.); 3 Department of Paediatric Haematology, Emma Children’s Hospital, Amsterdam UMC, University of Amsterdam, 1105 AZ Amsterdam, The Netherlands; 4 Academic Pediatric Center Suriname, Academic Hospital Paramaribo, Paramaribo, Surinameajuliana@azp.sr (A.J.); 5 University of Groningen, University Medical Centre Groningen, Beatrix Children’s Hospital, Department of Paediatric Gastroenterology, Hepatology and Nutrition, 9713 GZ Groningen, The Netherlands; p.f.van.rheenen@umcg.nl

**Keywords:** sickle cell disease, SCD, newborn screening, Suriname, non-adoption, abandonment, scale-up, spread and sustainability framework, NASSS, implementation research

## Abstract

The early detection of sickle cell disease (SCD) is vital to reduce mortality among affected children. Suriname currently lacks a newborn screening programme (NSP) for SCD. We performed a pilot programme to evaluate the scalability of such an initiative. Dried blood spots were collected from five birth centres and subjected to electrophoresis analysis. The programme scalability was evaluated using the non-adoption, abandonment, scale-up, spread, and sustainability framework. Challenges across six domains (illness, technology, value proposition, adopter system, organisation, and societal system), were categorised hierarchically as simple 😊, complicated 😐, or complex 😢. It has been proven that implementing programmes with mainly complicated challenges is difficult and those in mainly complex areas may be unachievable. SCD was detected in 33 of 5185 (0.64%) successfully screened newborns. Most of the domains were classified as simple or complicated. Disease detection and technology suitability for screening in Suriname were confirmed, with favourable parental acceptance. Only minor routine adjustment was required from the medical staff for programme implementation. Complex challenges included a reliance on external suppliers for technical maintenance, ensuring timely access to specialised paediatric care for affected newborns, and securing sustainable financial funding. Scaling up is challenging but feasible, particularly with a targeted focus on identified complex challenges.

## 1. Introduction

Sickle cell disease (SCD) is an inherited lifelong condition impacting haemoglobin, with potentially fatal consequences. A recent study on child mortality associated with untreated SCD in sub-Saharan Africa showed mortality rates of 43.3% for those younger than 10 years [1]. The early detection and treatment of SCD is vital to reduce mortality in affected children. The introduction of a newborn screening programme (NSP) for SCD and enrolment in a comprehensive treatment programme, involving the start of prophylactic penicillin between the ages of 2 and 4 months and parental education, has been shown to reduce mortality in affected children in both developed as well as in developing countries [2,3,4,5]. These interventions are especially of added value when initiated before the onset of symptoms.

SCD is most common among people of African, Indian, or Mediterranean ancestry. Migration has raised the incidence of SCD in the Americas. The prevalence of SCD in the Caribbean and the northern Atlantic coast of South America is between 0.26% and 0.65% [6]. Suriname, situated on the Northern Atlantic coast of South America, is classified as a middle-income country.

Suriname’s ethnic diversity is a legacy of its colonial history and migrations. The population comprises Indian-Surinamese (27.4%), Maroons (21.7%), Creoles (15.7%), Javanese–Surinamese (13.7%), individuals of mixed heritage (13.4%), and other groups (7.6%) [7]. Maroons are descendants of enslaved West and Central Africans who escaped from plantations in the 17th and 18th centuries. Creoles are typically of mixed African and European descent. Indian-Surinamese and Javanese-Surinamese individuals descend from labourers brought from India and Java in the late 19th and early 20th centuries.

The country’s birth rate is estimated to be around 9500 births per annum, with the majority occurring in hospitals or primary healthcare facilities [7]. Data on health literacy in Suriname are scarce. One study concluded that the health literacy was moderate [8]. The healthcare system in 2016 included five hospitals, all located on the coastal plain, of which four were in the capital, Paramaribo. Approximately 90% of the population lives on the Atlantic coastal plain and receives healthcare from regional primary health clinics. The population that lives in the sparsely populated non-coastal area receive primary healthcare services through clinics run by the Medical Mission [9].

Before the initiation of our pilot programme, Suriname lacked an NSP for any diseases, including SCD. There was no consensus on the concentration of specialised sickle cell disease services, nor were there national guidelines for a comprehensive treatment programme. The diagnosis of children with SCD occurs upon the manifestation of medical symptoms, some of which could be life-threatening. Currently, life expectancy data for SCD patients in Suriname are unavailable, and the percentage of children with SCD reaching adulthood remains unknown. Recognising the challenges and feeling ill equipped to manage children with advanced SCD symptoms, a group of paediatricians and clinical chemistry laboratory personnel took the initiative to establish a pilot NSP for the early detection of SCD. A multicentre pilot NSP for SCD, the first ever in Suriname, was conducted. The non-adoption, abandonment, scale-up, spread, and sustainability (NASSS) framework was used to assess the feasibility of scaling up the programme beyond the initial sites. The NASSS framework is an evidence-based framework that helps predict and evaluate the success of a technology-supported health programme and it creates a multidimensional overview of the context in which the programme is being implemented [10].

## 2. Materials and Methods

### 2.1. Outline of the NSP Pilot for SCD

Before the start of the NSP pilot for SCD, a paediatric working group was established to formulate treatment guidelines for children with SCD. These guidelines encompass various aspects, including the imperative to enrol children with SCD in a comprehensive treatment programme within specialised paediatric care. This programme includes initiating prophylactic penicillin between the ages of 2 and 4 months, along with parental education. The guideline was disseminated to both general practitioners (GPs) and paediatricians.

The NSP pilot for SCD operated in five birth centres from August 2015 to July 2016, involving the four largest hospitals in Paramaribo, Suriname, and the largest primary healthcare clinic of the Medical Mission. Midwives, gynaecologists, and primary healthcare providers of the participating birth centres were informed about the pilot and, in turn, informed pregnant women who came for antenatal care through both spoken and written information. No financial contribution was required for participation. Dried blood spots (DBSs) were obtained from newborns with parental consent, excluding those who received an erythrocyte transfusion before DBS collection was possible. In anticipation of future NSP expansion to incorporate additional diseases such as congenital hypothyroidism, our objective was to ensure DBS collection occurred no less than 24 h after birth. Our research protocol specifies that the timing of DBS collection varies across different birth centres, depending on the existing healthcare infrastructure, as outlined in Table 1.

Demographic data and contact information of the parents and the GPs were recorded on the information sheet attached to the DBSs. DBSs were transported to the core laboratory within seven days and analysed twice a week using capillary electrophoresis (CE) (Capillrys 2 Neonat Fast™, Sebia, France) following the manufacturer’s instructions. Repeat analysis of the same DBS sample was conducted for positive or indeterminate results. Positive SCD results triggered immediate phone notifications to parents and the GP for referral to specialised paediatric care. Efforts included persistent attempts to reach parents by phone, with multiple calls made if necessary. Additionally, follow-up calls were conducted after sharing the diagnosis with parents to ensure they successfully accessed specialised paediatric care. Uninterpretable results prompted phone contact with parents for repeat DBS collection. Negative results or carrier states for haemoglobinopathy were not disclosed to parents.

### 2.2. Ethical Considerations

The study protocol was reviewed and approved by the Human Research Ethics Committee of Suriname (ref: VG013-15). The committee recommended non-disclosure of data concerning carrier states for haemoglobinopathy.

### 2.3. Evaluation

**Figure 1:**
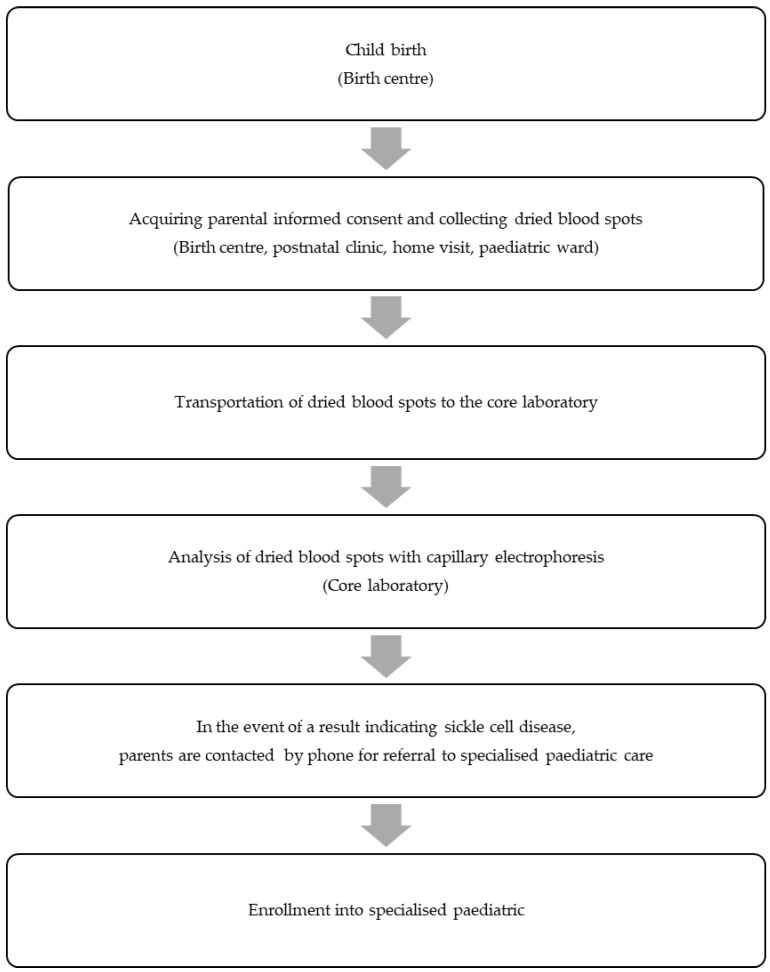
Successive steps in the newborn screening programme pilot for sickle cell disease that have been evaluated according to the non-adoption, abandonment, scale-up, spread, and sustainability framework.

During the NSP pilot, we compiled data from the information sheets and conducted semi-structured interviews with parents and the involved medical staff. Our assessment encompassed programme coverage and the entire process chain, spanning from the birth of a newborn to the point where the affected newborn enters specialised paediatric care (Figure 1).

To systematically explore challenges and determine the potential for upscaling the NSP for SCD to a nationwide level, we retrospectively applied the NASSS framework [3]. This framework comprises measures categorised into seven domains: (1) the illness, (2) the technology, (3) the value proposition, (4) the adopter system, (5) the organisation, (6) the societal system, and (7) the time dimension. For local applicability, we made slight adjustments to the questions (Table 2) and omitted the ‘time dimension’ domain. Following the methodology outlined in the original publication, we classified the measures hierarchically as simple 😊, complicated 😐, or complex 😢. Programmes with multiple complicated domains have proven challenging to implement, while those with multiple complex domains may not even become mainstream.

## 3. Results

### 3.1. NSP Pilot for SCD

**Figure 2:**
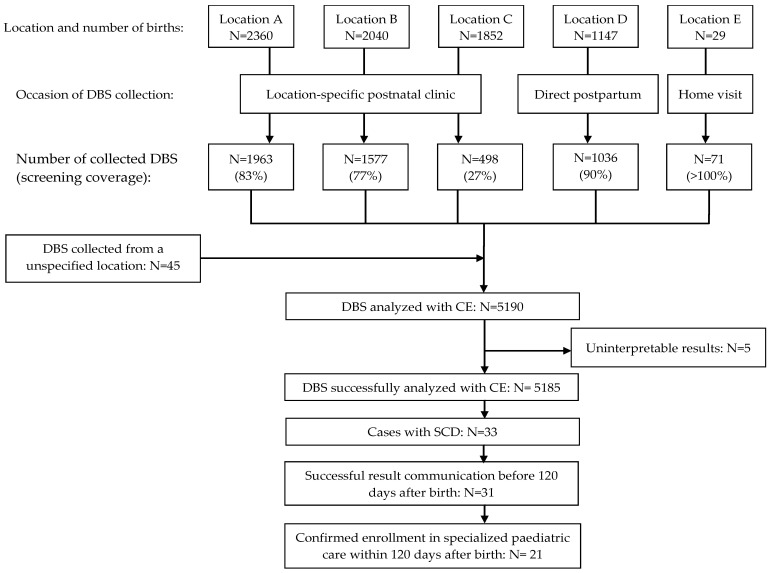
Flow diagram of the newborn screening programme pilot for sickle cell disease in Suriname.

During the NSP pilot, 7428 newborns were born in the participating birth centres (Figure 2). The average screening coverage was 69.9% and varied among birth centres, ranging from 27 to 100%.

A total of 5190 DBSs were collected and analysed by CE. Five cases yielded uninterpretable test results. SCD was identified in 33 out of the 5185 successfully screened newborns (0.64%; 95% confidence interval 0.45 to 0.89%). The demographic characteristics of all the screened newborns and those with SCD are detailed in Table 3. The predominant genotype was haemoglobin SC disease (HbSC, *n* = 17), followed by haemoglobin SS/S Beta-0 thalassemia disease (HbSS/HbSβ0 *n* = 15). One case involved haemoglobin SE disease (HbSE). Four instances of haemoglobinopathy other than sickle cell were identified (three cases of haemoglobin C disease (HbCC) and one case of alpha thalassaemia). We effectively communicated the result within 120 days after birth in 31 out of 33 cases (94%). Among the 33 affected newborns, 21 (64%) received specialised paediatric care within 120 days of birth. In seven cases, specialised paediatric care commenced between 120 and 160 days after birth. For five cases, attempts to contact parents by phone for follow-up and confirmation of specialised paediatric care were unsuccessful.

### 3.2. The Systematic Evaluation of Challenges of Implementing NSP for SCD Using the NASSS Framework

A summary of the challenges of implementing the NSP for SCD is given in Table 4. In the following sections, we give a more detailed overview of the considerations we have made during the rating process.

#### 3.2.1. Domain 1: The Illness

SCD can lead to potentially life-threatening complications. One of these complications is the entrapment of sickled red blood cells in the spleen, causing hyposplenism. Hyposplenism is present in the majority of children with SCD before the age of 12 months [11]. Hyposplenic individuals are immunocompromised and especially susceptible to invasive infections with encapsulated bacteria [12,13]. The main cause of mortality in young children with untreated SCD is invasive infections with a peak incidence of mortality between the age of 1 and 3 years [13]. A landmark study by Gaston et al. showed that penicillin prophylaxis reduced the incidence of infection in children with SCD by 84% [14]. Prophylactic penicillin as a measure to reduce infection and infection-related mortality in young children with SCD is recommended internationally [15,16].

The introduction of an NSP for SCD and enrolment in a comprehensive treatment programme, including the start of prophylactic penicillin between the ages of 2 and 4 months and parental education, has been shown to reduce mortality in affected children in both developed as well as in developing countries [2,3,4,5]. These interventions are especially of added value when initiated before the onset of symptoms. We classified this measure as simple 😊.

Social and cultural factors, including health literacy, could impact the traceability and healthcare-seeking behaviour of parents with newborns who are affected. However, the specific manner and extent of this influence remain indeterminate based on our study. In 2014, universal access to basic health insurance was introduced for all residents of Suriname. This insurance encompasses essential coverage for healthcare services integral to a comprehensive treatment programme for SCD. Nonetheless, as of the present time, an NSP is not available in Suriname. Consequently, the cost associated with the initial screening is presently not covered under basic health insurance. Should parents be required to contribute financially towards NSP expenses, the probability of participation may diminish due to economic obstacles. Therefore, the prospective implementation of NSP as a nationwide initiative is, to some extent, reliant on the financial framework supporting NSP. This measure was classified as complicated 😐.

#### 3.2.2. Domain 2: The Technology

Several studies have affirmed that CE is a reliable method for NBS for SCD [17,18,19]. In our pilot programme, the collected DBSs were transported to the core laboratory, equipped with the CE system. We encountered various technical, logistical, and communication challenges. In seven cases (0.1%), the results after CE analysis were uninterpretable, either due to an insufficient quantity of blood on the filter paper (N = 5) or analytical problems (N = 2). Attempts were made to collect new DBSs from these newborns for repeat testing, but only four DBSs could be obtained. Unfortunately, two out of these four DBSs were again deemed invalid due to insufficient blood quantity on the filter paper. Two cases were successfully retested. Addressing the missed opportunity to perform NBS in the case of uninterpretable results appears to be challenging.

The collected contact data were frequently incomplete, making it difficult to trace parents by phone. Oftentimes, several attempts were necessary before successful contact could be established. We classified this measure as complicated 😐.

The haemoglobin within the DBS undergoes separation into distinct fractions through CE. Following this separation, these fractions are automatically identified and presented in an electropherogram. The electropherogram proved to be easily interpretable by the trained local laboratory analyst.

In our pilot programme, we observed that the knowledge required to obtain and transport the DBSs was successfully transferred to the local medical staff. After training, the laboratory analysts could perform CE analysis independently. We classified measures 2B and 2C as simple 😊.

The CE technology relies on an external supplier for both technical maintenance and the provision of laboratory consumables. Throughout our pilot programme, we encountered technical errors that required resolution by the supplier’s technical personnel, who had to travel from abroad. This led to a few weeks of interruption in the analysis process, highlighting the system’s vulnerability to potential withdrawal by the supplier. Measure 2D was classified as complex 😢.

#### 3.2.3. Domain 3: The Value Proposition

We have observed that when NBS was provided in a highly accessible manner (immediately postpartum or during a postnatal home visit), parental consent was almost always obtained, resulting in a high screening coverage (90–100%). From a parental perspective, NBS for SCD is seen as a desirable form of preventive healthcare.

We classify uninterpretable results and failure to timely contact the parents for results communication as a potential safety risk. In our pilot programme, this occurred, respectively, in 7 cases out of the 5190 received DBSs (0.1%) and in 2 out of the 33 cases (6%) with positive screening results. With 21 of the 33 detected cases (64%) entering specialised paediatric care within 120 days after birth, we acknowledge that the efficacy is suboptimal and requires improvement.

Currently, there are limited data on the cost-effectiveness of NBS for SCD in the Caribbean region. A study that assesses the cost-effectiveness of an NBS and a comprehensive treatment programme for SCD in 47 sub-Saharan African countries concluded that NBS for SCD is estimated to be cost-effective as long as the incidence rate exceeds 0.2–0.3%. However, in some countries, NBS is cost-effective at incidence rates below this range [20]. The incidence rate we observed in our pilot was 0.64%, surpassing the balance point of cost-effectiveness by more than twofold.

Evaluation of the various challenges of domain 3: The value proposition shows that most of the challenges (desirability, safety, and cost-effectiveness) can be classified as simple or complicated. However, the challenge efficacy needs improvement and is therefore classified as complex. We classified this measure in its entirety as complicated 😐.

#### 3.2.4. Domain 4: The Adopter System

In our pilot programme, the majority of the medical staff needed to acquire relatively straightforward new skills, while a minority required training in more complex tasks, such as conducting CE analysis. All the new tasks fit into their existing professional identities.

In three out of the five birth centres, DBSs were collected during postnatal clinic visits. In these settings, parents were expected to bring their newborn to the birth centre to participate in the NSP. However, this was not feasible for all parents. The intended procedure involves parents of affected newborns being contactable by phone for result communication, followed by prompt contact with their GP for referral to specialised paediatric care. However, in certain instances, parents were difficult to reach by phone, and some required multiple reminders before visiting their GP.

Measure 4A was classified as simple 😊, while 4B was deemed complex 😢.

#### 3.2.5. Domain 5: The Organisation

In our pilot programme, we collaborated with five healthcare institutions, collectively referred to as the organisation within this context. The engagement with these institutions was marked by a blend of enthusiasm for innovation and apprehension towards change. However, as in most resource-limited settings, innovation was not a top priority. The involvement of local medical staff is crucial in maximising screening coverage. One particular birth centre exhibited a notably low screening coverage (27%) compared to the other birth centres (77–100%), likely due to fluctuating levels of engagement among local staff and insufficiently informed personnel resulting from staff rotations caused by shortages. Consequently, both measures 5A and 5B were classified as complicated 😐.

Our pilot programme was funded by a project-based donation. Consequently, securing a new funding strategy is imperative for sustaining and expanding the NSP. While much of the infrastructure required for our pilot programme was repurposed from existing resources, funding was necessary for personnel costs, laboratory tests, and equipment maintenance. This aspect was categorised as complex 😢.

Minor adjustments were made to team routines and care pathways, ensuring alignment with existing ones. For instance, a phlebotomist was incorporated into the staff of the postpartum outpatient clinics at locations A, B, and C, facilitating the efficient collection of DBSs without disrupting existing care pathways. This initiative was deemed simple 😊.

Throughout our pilot programme, consistent oversight from a dedicated small team was essential to maintain engagement and training among medical staff, manage logistics and supplies, and assess and enhance the NSP. A similar team will be essential to lead the implementation of a national NSP in Suriname. This aspect was classified as complex 😐.

#### 3.2.6. Domain 6: The Wider Context

At the outset of our pilot programme, the Ministry of Health of Suriname had published plans to improve the efficiency and accessibility of healthcare in the country. The primary strategy highlighted the importance of investing in preventive healthcare. Therefore, the NSP for SCD aligns with this political vision.

Economic fluctuations in Suriname significantly impact the healthcare system. During periods of economic uncertainty, resources are typically directed towards acute and curative healthcare objectives. Consequently, the sustainability of a national NSP for SCD in Suriname is partially contingent on the economic climate.

In Suriname, the medical referral system mandates that specialised paediatric care requires a referral from a GP for enrolment. The delay in enrolling affected newborns into specialised paediatric care is partly attributed to referral delays. In three instances, parents clarified that the delay in enrolment following a referral was due to the newborn not being registered with a health insurance plan. Although basic health insurance is available to all Surinamese residents, newborns must be formally registered to access its benefits. In summary, the current medical referral system in Suriname is not yet optimised to facilitate the timely enrolment of affected newborns into specialised paediatric care. This measure was classified as complicated 😐.

## 4. Discussion

During our 10-month pilot programme, we successfully screened 5186 newborns for SCD. The results of our pilot programme reveal a high prevalence of SCD in Suriname (0.64%).

The evaluation of the pilot programme using the NASSS framework showed that most measures could be classified as simple or complicated, while three measures were deemed complex. This indicates that further implementation of the programme will be difficult but not impossible.

An NSP for SCD in Suriname is technically feasible and an acceptable form of preventive care from the perspective of parents. No significant alterations were necessary in the current healthcare system to implement the pilot programme, except for the referral process, which requires adaptation to better align with the goals of the NSP. Significant challenges identified include reliance on an external supplier for the technical maintenance of the CE system and laboratory consumables, ensuring timely access to specialised paediatric care for all affected newborns, and securing sustainable financial funding for the programme’s ongoing implementation.

Precise numbers on the prevalence of SCD in Suriname have not been reported previously. In our pilot programme, the incidence of SCD is 6.36 per 1000 screened newborns, which is comparable to rates in other Caribbean countries [6]. Contrastingly, in the Netherlands and the UK, where an NSP for SCD is nationally implemented, the incidence is notably lower, at 0.127 and 0.329 per 1000 screened newborns, respectively [21,22]. Additionally, the occurrence of diseases that are included in NSPs globally, such as congenital hypothyroidism (0.33 per 1000) and galactosemia (0.02 per 1000), is also significantly lower [23,24].

SCD should be recognised as a very common disease in Suriname, and there is potential to prevent its life-threatening health complications through early detection. This justifies the implementation of an NSP for SCD in Suriname [25]. In our pilot programme, SCD was identified in newborns from various ethnic backgrounds, indicating that a universal screening approach would be suitable for Suriname.

The efficiency of the NSP is significantly influenced by the extent of screening coverage and the prompt communication of results. Challenges in suboptimal screening coverage and result communication have been reported in multiple other studies in developing countries [26,27].

The screening coverage may be influenced by factors related to both organisational aspects and barriers to participation. The three centres with the lowest screening coverage all collected DBS samples during postnatal clinic visits. In contrast, centres that collected DBS samples immediately after birth and during postpartum home visits demonstrated a significantly higher screening coverage. One potential explanation for this disparity could be the elimination of the need to attend a postnatal clinic with the newborn to participate in the NBS. We were surprised to observe that the birth centre that serves the population of the non-coastal area and offered the NSP during a home visit 7 days postpartum had a screening coverage of >100%. We hypothesise that postpartum migration and home births might account for this observation.

The screening coverage might be optimised by screening directly postpartum, during a postpartum home visit or at the time of the first immunisation. In Suriname, the initial immunisation is administered by a GP when the newborn reaches two months old, with a ≥90% coverage reported in the last six years [28]. This approach of conducting newborn screening directly after childbirth or during the first immunisation may be appropriate for SCD screening. Nevertheless, the timing may not be ideal for detecting other conditions that are typically part of an NSP. Certain diseases can only be screened for after the initial days of life, and there are instances where medical interventions are required before the child reaches two months of age.

New point-of-care tests for SCD screening are currently in development [29,30]. These tests have the potential to revolutionise diagnostics because they provide rapid test results that can be immediately communicated to parents. The success of the new generation of tests will depend on factors such as cost, availability, and acceptance by frontline healthcare staff.

Strengths and weaknesses

The primary strength of our study lies in the comprehensive inclusion of the major birth centres in Suriname, ensuring that over 50% of the annual livebirth newborns were offered the NSP during the study period of ten months. Moreover, the NASSS method offers a highly multidimensional perspective across various domains, enhancing our confidence in providing a thorough overview of the challenges anticipated in the national implementation of an NSP for SCD in Suriname.

The effectiveness of an NSP for SCD is underscored by its potential to prevent mortality and morbidity among young patients. However, due to constraints relating to cost and time, our study primarily focused on enrolment in specialised paediatric care at 120 days after birth as the primary outcome, rather than directly capturing mortality and morbidity outcomes. This represents a significant limitation of our study.

Additionally, the retrospective application of the NASSS method has posed a constraint. Had we applied the NASSS method prospectively, we could have seized opportunities to enhance our pilot programme in real time. For instance, we could have developed alternative strategies to ensure timely access to specialised paediatric care for all affected newborns.

Implications

The gathered data on the prevalence of SCD in Suriname affirm its status as a highly prevalent condition in the country. These data aid Suriname’s health policymakers in making well-informed decisions regarding health policy. An analysis using the NASSS framework highlights the significant challenges involved in implementing an NSP for SCD in Suriname. As the initiative advances towards national implementation, key priorities should include addressing dependence on external suppliers for technical maintenance, ensuring the prompt inclusion of all affected newborns in specialised paediatric care, and securing sustainable funding.

Recommendations

We propose the introduction of free universal screening for SCD for infants under the age of three months in Suriname. Depending on the feasibility of initiating an NSP for other prevalent diseases, options include replicating the NSP procedures from our pilot programme or exploring the potential for point-of-care (POC) screening immediately postpartum or during the initial immunisation.

## 5. Conclusions

SCD is a potentially life-threatening disease that is highly prevalent in Suriname. The early detection and treatment of SCD can significantly diminish childhood morbidity and mortality. Scaling up the NSP for SCD in Suriname will present challenges, yet it remains feasible with a concerted effort.

## Figures and Tables

**Table 1:** Timing and environment of DBS collection per location.

Location	Timing of DBS Collection	Environment	Motivation
Birth centres A, B, C	7–14 days after birth	Postnatal clinic	Postpartum discharge was usually ≤24 h after birth
Birth centre D	Directly postpartum	Obstetric ward	Absence of standard postnatal clinic
Birth centre E	7 days after birth	Home visit	Discharge was usually ≤24 h after birth
Paediatric ward of locations A, B, C, D	24 h after birth	Paediatric ward	Need for newborn monitoring

Note. Birth centres A, C, and D are general hospitals. Birth centre B is a tertiary hospital. Birth centre E is a primary healthcare clinic.

**Table 2:** Overview of the domains and questions in the non-adoption, abandonment, scale-up, spread, and sustainability framework and the adjusted questions we used for local applicability.

Domain and Questions	Adapted Questions
Domain 1: The illness	
1A. What is the nature of the condition or illness?	Does newborn screening for sickle cell disease result in health gain?
1B. What are the relevant sociocultural factors and comorbidities?	What are potential sociocultural factors that could interfere with participation in the newborn screening programme?
Domain 2: The technology	
2A. What are the key features of the technology?	No changes
2B. What kind of knowledge does the technology bring into play?	No changes
2C. What knowledge and/or support is required to use the technology?	No changes
2D. What is the technology supply model?	No changes
Domain 3: The value proposition	
3A. What is the developer’s business case for the technology (supply side value)?	Not applicable
3B. What is its desirability, efficacy, safety, and cost-effectiveness (demand-side value)?	No changes
Domain 4: The adopter system	
4A. What changes in staff roles, practices, and identities are implied?	No changes
4B. What is expected of the patient (and/or immediate caregiver)? Is this achievable by and acceptable to them?	What is expected of the parents of the newborn—is this achievable by them?
4C. What is assumed about the extended network of lay caregivers?	Not applicable
Domain 5: The organisation	
5A. What is the organisation’s capacity to innovate?	No changes
5B. How ready is the organisation for this technology-supported change?	No changes
5C. How easy will the adoption and funding decision be?	No changes
5D. What changes will be needed in team interactions and routines?	No changes
5E. What work is involved in implementation and who will do it?	No changes
Domain 6: The wider context	
6A. What is the political, economic, regulatory, professional (e.g., medicolegal), and sociocultural context for programme rollout?	What is the political, economic, and regulatory context for programme rollout?
Domain 7: The time dimension	
7A. How much scope is there for adapting and coevolving the technology and the service over time?	Not included
7B. How resilient is the organisation to handling critical events and adapting to unforeseen eventualities?	Not included

**Table 3:** Demographic characteristics of all screened newborns and newborns with sickle cell disease.

		All Screened Newborns (N = 5190)	Newborns with Sickle Cell Disease (N = 33)
Gender	Female	2533 (48.7%)	23 (69.7%)
	Male	2583 (49.8%)	10 (30,3%)
	Missing	74 (1.4%)	0
Ethnicity	Maroons	1333 (25.7%)	12 (36.4%)
	Creoles	1171 (22.6%)	10 (30.3%)
	Mixed ethnic background	982 (18.9%)	8 (24.2%)
	Indian–Surinamese	766 (14.8%)	0
	Javanese–Surinamese	463 (8.9%)	0
	Chinese–Surinamese	134 (2.6%)	0
	Indigenous	138 (2.7%)	2 (6.1%)
	Missing	203 (3.9%)	1 (3.0%)

**Table 4:** Summary of the systematic evaluation of challenges of implementing the newborn screening programme for sickle cell disease using the non-adoption, abandonment, scale-up, spread, and sustainability framework.

Domain and Questions	Rating	Comment
Domain 1: The illness		
1A. Does newborn screening of sickle cell disease result in health gain?	😊	Newborn screening of sickle cell disease and enrolment in a comprehensive treatment programme has been shown to reduce mortality in affected children.
1B. What are potential sociocultural factors that could interfere with participation in the newborn screening programme?	😐	Social and cultural factors, including health literacy, could impact the traceability and healthcare-seeking behaviour of parents with newborns who are affected. The potential demand for parental financial contribution could be a barrier to participating in the newborn screening programme.
Domain 2: The technology		
2A. What are the key features of the technology?	😐	Capillary electrophoresis was carried out in the core laboratory. This caused logistical challenges.
2B. What kind of knowledge does the technology bring into play?	😊	Interpretation of the electropherogram was straightforward.
2C. What knowledge and/or support is required to use the technology?	😊	The knowledge to obtain the dried bloodspot and to perform the laboratory analysis was successfully transferred to the local medical staff.
2D. What is the technology supply model?	😢	Capillary electrophoresis technology is vulnerable to supplier withdrawal.
Domain 3: The value proposition		
3A. Not applicable		
3B. What is its desirability, efficacy, safety, and cost-effectiveness (demand-side value)?	😐	From the parent’s point of view, newborn screening for sickle cell disease is a desirable form of preventive healthcare. Potential safety risks include uninterpretable results and failure to contact parents timely for results communication.
Domain 4: The adopter system		
4A. What changes in staff roles, practices, and identities are implied?	😊	Small changes in the staff roles and practices were needed, all appropriate to the existing professional identities.
4B. What is expected of the parents of the newborn? Is this achievable by them?	😢	In birth centre locations A, B, and C, parents needed to bring their newborn to the postnatal clinic to participate in the newborn screening programme. Parents of affected newborns were expected to be traceable by phone. Both expectations were not always met by parents.
4C. Not applicable		
Domain 5: The organisation		
5A. What is the organisation’s capacity to innovate?	😐	The organizations were enthusiastic about innovation; however, as in most middle-income countries, due to structural severe resource pressures, innovation is not a top priority.
5B. How ready is the organisation for this technology-supported change?	😐	
5C. How easy will the adoption and funding decision be?	😢	A new strategy for funding needs to be developed when needed when the continuation or upscaling of the NSP will be taking place.
5D. What changes will be needed in team interactions and routines?	😊	Small changes in the team routines were implemented.
5E. What work is involved in implementation and who will do it?	😐	A small dedicated team will be required to manage the newborn screening programme.
Domain 6: The wider context		
6A. What is the political, economic, and regulatory context for programme rollout?	😐	Investment in preventive healthcare, such as newborn screening, corresponds to the health sector development plan of the Ministry of Health of Suriname. However, during economically uncertain times, the available resources will most likely be allocated for acute and curative healthcare goals. The current medical referral system in Suriname is not optimised to support the timely enrolment of the affected newborns into specialised paediatric care.
Domain 7: The time dimension		
7A. Not applicable		
7B. Not applicable		

## Data Availability

The data supporting reported results can be found at https://osf.io/ma2ht/?view_only=5a10e0fed3b74daa90998d8167f6ea1b (accessed on 30 June 2024).
